# Development of a Boron Nitride-Filled Dental Adhesive System

**DOI:** 10.3390/polym15173512

**Published:** 2023-08-23

**Authors:** Senthilguru Kulanthaivel, Jeremiah Poppen, Sandra Ribeiro Cunha, Benjamin Furman, Kyumin Whang, Erica C. Teixeira

**Affiliations:** 1 Department of Operative Dentistry, College of Dentistry & Dental Clinics, The University of Iowa, Iowa City, IA 52242, USAjeremiah-poppen@uiowa.edu (J.P.);; 2 Southwest Research Institute, San Antonio, TX 78238, USA; 3 Department of Comprehensive Dentistry, The University of Texas Health Science Center at San Antonio, San Antonio, TX 78229, USA; whang@uthscsa.edu

**Keywords:** boron nitride, nanofillers, dental adhesives, water sorption, mechanical properties

## Abstract

There is a dearth of adhesive systems capable of forming stable bonds between restorative materials and tooth surfaces. To address the concern, this study determined the effects of using methacrylate-functionalized boron nitride nanosheets (BNNSs) in a polymeric dental adhesive system. The bisphenol A glycidyl dimethacrylate (BisGMA):2 hydroxyethyl methacrylate (HEMA) (60:40) adhesive monomer blend with a photoinitiator was filled with 0 wt% (control), 0.1 wt%, and 1 wt% BNNSs and light cured. Fourier transform infrared spectroscopy was performed to determine the conversion degree of monomer double bonds (DoC). Water absorption and solubility were measured. Flexural strength and Youngs’s modulus were evaluated to determine the mechanical properties of the composite adhesive system. Finally, dentin bond strength degradation and fracture mode were quantified with a microtensile bond test to confirm the bonding ability of the developed adhesive system. Results showed that the incorporation of BNNSs increased DoC (9.8% and 5.4% for 0.1 and 1 wt%, respectively), but it did not affect water sorption (101.9–119.72 (µg/mm^3^)), solubility (2.62–5.54 (µg/mm^3^)), Young’s modulus (529.1–1716.1 MPa), or microtensile bond strength (46.66–54.72 MPa). Further studies are needed with varying BNNS loading percentages from 0.1 wt% to 1 wt% in order to more comprehensively determine the effect of BNNSs on dental adhesives.

## 1. Introduction

Over the past decade, adhesive dentistry has witnessed a significant advancement in materials’ compositions and their adoption rate. However, in the case of broad polymer-based adhesive systems, the reduction in bond strength, water absorption, interfacial and marginal degradation, and biocompatibility still pose great challenges to the success of these materials [1,2]. The complex nature of the dentin—i.e., its hydrated inorganic (hydroxyapatite) and organic (collagen) parts and its dynamic wet tubular structures—provides a major challenge to the bonding materials [3,4,5]. The hydrophilicity of the monomers used in dental adhesives leads to high water absorption at the interface [6]. The infiltrated water present in the organic matrix of dentin may not be replaced completely by hydrophilic monomers such as HEMA, which even compromises the dentin sealing effect. On the other hand, hydrophobic monomers with di-methacrylate’s such as BisGMA, Urethane di-methacrylate (UDMA), etc., could form a strong polymeric network but are limited by their low solubilities in the water [7,8]. Thus, these materials fail to withstand the dynamic physical, chemical, and mechanical stress caused at the interface [9]. Hence, research has been focused on exploring different strategies with which to improve the properties of dental adhesive materials. 

One promising approach to combat these shortcomings is the incorporation of filler materials into the adhesive system to increase bond strength and longevity by decreasing water sorption and hydrolytic degradation rate [10]. These filler materials play a vital role in forming a strong intricate hybrid layer between the tooth surface and the resin monomers [11,12]. However, the filler size and quantity affect the desired outcome [13]. Decrease in primary particle size generally increases adhesive penetration into the tubules. Several studies have shown that reducing the size of filler materials from micron to nanoscale could improve the mechanical properties of the adhesives [14,15]. Apart from size, the composition of the bonding agents might influence the tooth–material interface. For some bonding agents, secreted ions could interact with the dentin layer affecting the bonding strength [7,16]. On the other hand, an increase in filler loading could reverse the benefit of filler addition by increasing the viscosity of the material and decreasing filler infiltration into the demineralized organic dentin matrix. This, in turn, could affect the kinetics of the adhesive monomer’s polymerization and ultimate degree of conversion [17]. 

In this regard, several studies focused on the development of nanofillers-based dental adhesives with improved properties [18,19,20,21,22]. Amongst different nanomaterials, two-dimensional nanosheets, especially graphene, an allotrope of carbon arranged in a single atomic hexagonal layer, has been of interest due to its high strength and modulus (0.5 TPa) [9,23,24,25,26]. However, graphene is not used in dentistry because it is dark-colored and expensive to manufacture. Meanwhile, boron nitride nanosheets (BNNSs), an analog to graphene known as “white graphene”, have shown promise in dentistry because hexagonal boron nitride (h-BN) is colorless in comparison to graphene. Similar to graphene, BNNSs can self-assemble into colloidal liquid crystals that have high strength, modulus, fracture toughness, and wear-resistance [27,28,29,30,31,32]. It is also chemically stable, biocompatible, and has low density [33,34,35,36]. Its lamellar structure may also reduce composite viscosity and, in turn, allow for an increase in filler loading [37]. It was reported that the loading of 0.3 wt% BNNSs increased the elastic modulus of polymethyl methacrylate films by ~11% [38]. In another study, 0.15 wt% loading of chemically comparable boron nitride nanotubes (BNNTs) into dental resins affected the contact angle, microhardness, and hydrolytic degradation strength. However, higher loadings of BNNTs might not be possible because they significantly increase resin blend viscosity [1,39,40]. 

Well-exfoliated BNNSs may potentially retain the benefits of BNNTs and be used at higher loadings without increasing the viscosity. However, shortcomings of current exfoliation methods, such as low yields and the production of damaged BNNS structures, limit their application in dentistry. In the current work, we hypothesized that the use of undamaged, exfoliated BNNSs could protect the filler–resin interface from hydrolytic degradation and decrease viscosity, allowing this degradation-resistant adhesive to infiltrate the collagen network more efficiently, thus increasing restoration longevity. Here, a modified method was used to exfoliate BNNSs without inducing any damage. These BNNSs were loaded into an adhesive monomer blend at different filler loadings (0, 0.1, and 1 wt%), and the adhesive degree of monomer-to-polymer conversion (Fourier transform infrared spectroscopy (FTIR)), water sorption, mechanical properties, and microtensile bond strength were determined. 

## 2. Materials and Methods

### 2.1. Materials

Bisphenol A glycidyl dimethacrylate (BisGMA), 2 hydroxyethyl methacrylate (HEMA), Camphoroquinone (CQ), Ethyl-4(dimethylamino) benzoate (EDMAB), and Diphenyliodonium hexafluorophosphate (DPIHP), 2-isocyanatoethyl methacrylate, and 2-butanone were procured from Sigma Aldrich, Inc. St. Louis, MO, US. Sodium hydroxide (NaOH) and methacryloxypropyl (trimethoxy) silane were procured from Fischer Scientific and Gelest Inc., Waltham, MA, USA, respectively.

### 2.2. Methods

#### 2.2.1. Exfoliation and Functionalization of BNNSs

**Figure 1:**
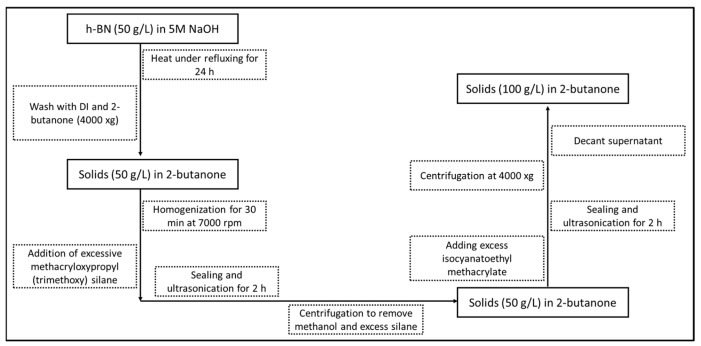
Schematic representation of reaction mechanism for Exfoliation and functionalization of BNNSs.

All reagents were used as received without further purification. The h-BN (50 g/L) was heated under refluxing conditions in a 5M aqueous solution of NaOH for at least 24 h (Figure 1). The solids were recovered using low-speed centrifugation (<4000× *g*, Avanti J-15R, Beckman Coulter, Chaska, MN) and washed sequentially with DI water and 2-butanone (Sigma Aldrich, St Louis, MO, USA). Wetted solids were dispersed in fresh 2-butanone (50 g/L) using rotor–stator (Fisherbrand 850, Fisher Scientific, Waltham, MA, USA) homogenization at 7000 rpm for 30 min. Following the homogenization, a large excess of methacryloxypropyl (trimethoxy) silane was immediately added to the 2-butanone suspension to drive condensation with the borated BNNS edges without a catalyst. The vessel was sealed, and the suspension was sonicated in an ultrasonic (Branson model IC1216) bath for at least 2 h. The solids were then centrifuged, removing both methanol and excess silane, and then resuspended in 2-butanone. Later, isocyanatoethyl methacrylate (Sigma Aldrich, St Louis, MO, USA) was added in excess to the suspension, sealed, and continued with sonication, substantially improving the suspension stability in 2-butanone. The resulting solids were settled by centrifugation at 4000× *g*, the reaction mixture was decanted, and the solids were resuspended (100 g/L) in 2-butanone prior to solvent/monomer exchange procedures. In general, the BNNS material was never allowed to dry. However, a small portion of the treated solids were tested by thermogravic analysis (Netzsch STA449), showing a mass gain of 3% versus the starting material.

#### 2.2.2. Adhesive Formulation

The adhesive monomer blend of 60:40 BisGMA: HEMA was filled with 0 wt% (control), 0.1 wt%, or 1 wt% methacrylate-functionalized BNNSs. A well-established three-component photoinitiator system was used: 0.5 wt% of camphoroquinone (CQ), 0.5 wt% ethyl-4(dimethylamino) benzoate (EDMAB), and 1 wt% diphenyliodonium hexafluorophosphate (DPIHP) [41]. 

#### 2.2.3. Monomer-to-Polymer Degree of Conversion (FTIR)

The conversion degree of monomer double bonds (DoC) of composites was determined using Fourier transform infrared spectroscopy (FTIR) (Nicolet 6700 FT-IR Fourier Transform Infrared Spectrometer). The DoC was determined using the aliphatic C=C peak at 1638 cm^−1^ with aromatic C–C peak at 1608 cm^−1^ as internal reference peak before and after curing [17,39,42,43]. The following formula (1) was used to calculate DoC:

(1) $$DoC\;\left(\%\right)=\left\{1-\left(\frac{\left(\;\frac{1638\;cm^{-1}\;}{1608\;cm^{-1}}\right)\;peak\;absorbance\;after\;curing}{\left(\frac{1638\;cm^{-1}}{1608\;cm^{-1}}\right)peak\;absorbance\;before\;curing}\right)\right\}*100.$$

#### 2.2.4. Water Sorption and Solubility

Disc-shaped specimens (Diameter: 15 ± 0.5 mm; Thickness: 0.9 ± 0.2 mm) were fabricated for each adhesive formulation. Disc-shaped specimens (diameter: 15 ± 0.5 mm; thickness: 0.9 ± 0.2 mm) were fabricated for each adhesive formulation. The adhesive formula was poured into a washer positioned on top of a clear plastic slide. Another plastic slide was placed on top of the washer then taken off to ensure adhesive maintained consistency with the washer’s depth. Each side was cured for 20 s with a Valo Cordless LED curing light (Ultradent Products, Inc., South Jordan, UT, USA). Initially, the light was positioned over the specimen’s center then repositioned about every 2 s in a circular pattern around the perimeter at 8 specific spots to follow ISO 4049. The washer was then flipped, the plastic slide removed, and the curing procedure repeated. The specimens were polished on a rotation polishing wheel with 400 then 800 grit abrasive paper. Once specimens were visibly and tactically smooth and the periphery abraded, specimens were post-processed at 23 +/− 2 °C and immersed in distilled water to prewash for 7 days. Subsequently, the specimens were dried in high vacuum temperature and then stored in a vacuum oven in the presence of dried silica at 37 °C. The dried specimens were weighed every 24 h until a constant mass had been reached (m1). Then, the dried specimens were soaked in distilled water. The samples were removed from the water at a fixed time point and the excess water was blotted against tissue paper. The samples were then weighed (m2) and incubated again in the water. This process was performed for the baseline measurement (7 days) and 12 months. Chloramine-T (0.5%) was replaced weekly to avoid contamination (ISO/DTS 11405 Test Type 3) [44]. For solubility, the samples were then dried as before until constant weight had been reached (m3). Water sorption was calculated using the following equation [17,45,46,47]:

Water sorption = ((m2 − m3)/V).(2)

Water solubility was calculated using the following formula:

Water Solubility = ((m1 − m3)/V),(3)

where V is the initial volume of the respective samples. 

#### 2.2.5. Flexural Strength and Young’s Modulus

Specimens were light-cured in a 2 mm × 2 mm × 10 mm Teflon mold between two glass slides and then light-cured at 1000 mW/cm^2^ for 20 s using an Ultradent Valo^®^ LED curing lamp. Then, the specimens were stored for 24 h at 37 °C and loaded in a universal testing machine (Zwick/Roell, Zwick GmbH and Co., Ulm, Germany) and tested using the three-point bending mode at 1 mm/min crosshead speed with a span of 8 mm until fracture [5]. The flexural strength and Young’s modulus values were obtained directly from the testXpert II–V3.71 testing software of the Zwick/Roell machine. 

#### 2.2.6. Morphological Analysis

To study surface morphology, we examined the outer surfaces of samples along with the fractured surfaces from flexural strength samples. The samples were vacuum dried overnight, and sputter coated with gold (K550 Emitech Sputter Coater) for 90 s at 10 mA. The samples were visualized with help of Hitachi S-400 SEM under vacuum of 3 KV and 10 µA. 

#### 2.2.7. Microtensile Bond Strength (µTBS) and Failure Pattern Analysis

Thirty sound, human third molars, less than 6 months from their extraction date, were mounted in dental stone by their notched roots. Middle dentin was exposed on the occlusal surface using a 55-carbide bur under copious air–water spray using an electric handpiece at 200,000 rpm in a custom-made cutting device (CNC Specimen Former: University of Iowa). A uniform substrate for dentin bonding was created. Randomly, teeth were assigned to the three experimental groups: 0 wt% (control); 0.1 wt%; 1 wt% BNNSs (*n* = 10). A commercial self-etch primer (Clearfil SE, Kuraray, Tokyo, Japan) was applied according to manufacturer’s instructions followed by the application of the experimental adhesive and light-cured by 20 s (Valo Grand, Ultradent, South Jordan, UT, USA). Build-ups of ~4 mm were made with a commercial composite resin (Filtek One Bulk Fill, 3M). Three 2 mm × 2 mm resin–dentin sticks were acquired from each tooth using an Isomet 1000 sectioning machine (Buehler, Lake Bluff, IL, USA). Each resin–dentin stick was further trimmed using the CNC machine into a dumbbell with a cross-sectional area of 0.5 mm^2^, gauge length of 1 mm, and radius of curvature of 0.6 mm. Specimens were stored in aqueous storage media containing 0.5% Chloramine-T at 37 °C for 24 h (baseline) and 6 and 12 months. The aqueous storage media containing 0.5% Chloramine-T was replaced weekly to avoid contamination (ISO/DTS 11405 Test Type 3). Dumbbells were tested at room temperature using a non-gluing passive gripping device (Dircks Device, University of Iowa, Iowa City, IA, USA) at a crosshead speed of 1 mm/min on a calibrated Zwick Materials Testing Machine (Zwick Gmbh and Co., Ulm, Germany). Each specimen was observed under a light microscope (Stemi 2000-C, Carl Zeiss, Oberkochen, Germany) with a magnification of 50×, and the failure mode was classified either as cohesive in dentin- or resin-based composite, adhesive, or mixed [17,48,49].

#### 2.2.8. Statistical Analysis

Statistical analysis was carried out using one-way ANOVA for the DoC, flexural strength, Young’s modulus, water sorption, and solubility studies. Repeated measures two-way ANOVA was used for µTBS study. A *p*-value of 0.05 or less was taken as significant. All the data were expressed as mean ± standard deviation. 

## 3. Results and Discussion

### 3.1. Degree of Conversion

**Figure 2:**
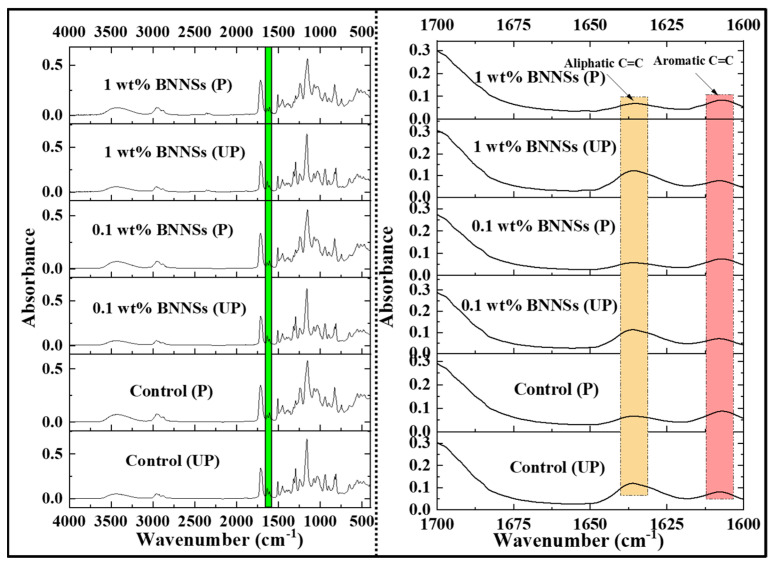
FTIR analysis of the unpolymerized (UP) and polymerized (P) adhesive samples (representative data).

**Figure 3:**
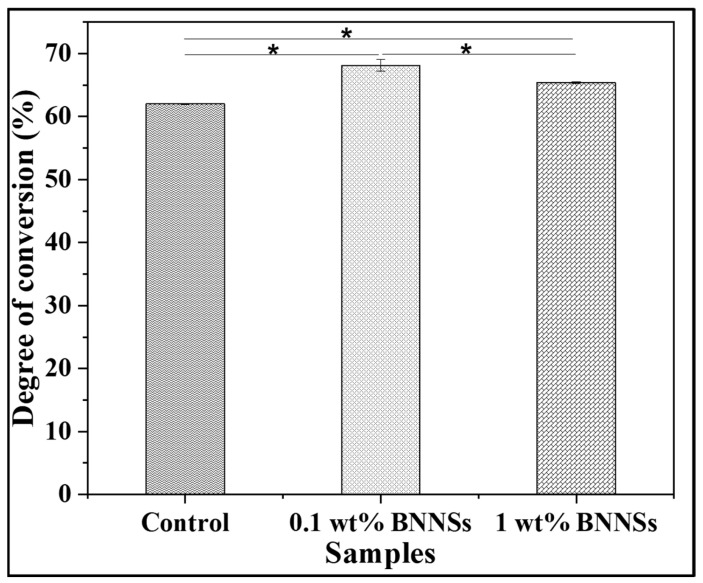
DoC (%) of the adhesives. The data were expressed as mean ± standard deviation. Statistical significance was evaluated for *p* < 0.05 via one-way ANOVA. * Data were statistically significant (*p* < 0.05).

Several studies have shown that the DoC is inversely proportional to filler loading [17,50]. Here, we have determined the degree of conversion using FTIR (Figure 2). On the contrary, we observed an increase in DoC with the addition of methacrylate-functionalized BNNSs (Figure 3) at low loading. The control (0 wt% BNNSs) had a DoC of about 62.02 ± 0.10%, whereas 0.1 wt% BNNSs had 68.11 ± 0.93% (*p* < 0.05). However, with further increase in the filler concentration, there was a decrease in the DoC, with 1 wt% BNNSs having a DoC of about 65.41 ± 0.15%. However, this was still significantly higher in comparison to the control (*p* < 0.05). As we hypothesized, methacrylate functionalization could improve the dispersion of the BNNSs in the polymer blend. Secondly, it can improve the chemical inertness of pristine BNNSs where there could be a favorable interaction of functionalized BNNSs with monomers. These could be the plausible reasons for increased DoC in case of 0.1 wt% BNNSs. As the concentration of fillers increases, the agglomeration of filler particles affects the penetration depth of the light and the scattering of light [48]. The same was observed in the current study, where the addition of BNNSs above 0.1 wt% BNNSs had a negative effect on DoC. However, the effect was not detrimental in comparison to control (0 wt% BNNSs). In a similar study, Degrazia et al. (2017) showed that with an increase in the addition of boron nitride nanotubes, the DoC increased until 0.1% (87.97 ± 1.65% for control and 91.74 ± 0.43% for 0.1% sample), but the addition of 0.15% of the nanotubes decreased the DoC (89.54 ± 1.29%) [1]. Similarly, in another study, AlRefeai et al. (2021) showed that the calcium-fluoride-nanofiller-added sample had a higher DoC (61.54 ± 4.07) in comparison to the control (56.8 ± 5.5) [51]. The study clearly showed that addition of BNNS fillers has increased the DoC significantly, which is highly desirable for any adhesive system. 

### 3.2. Water Sorption and Solubility

Water sorption and solubility are among the critical factors that affect the longevity of an adhesive system since higher water or solvent absorption weakens the matrix through swelling. This could also further lead to solubilization, degradation, and/or further buildup of mechanical stresses. The mechanical stresses could induce microcracks at the interface and cause the failure of the system [6,52,53]. Here, BNNSs were added as fillers to the current adhesive, hypothesizing that their intrinsic hydrophobicity could decrease water sorption and solubility, thereby increasing the longevity of the system/composite. 

The water sorption results showed that the addition of BNNSs did not significantly affect water sorption at baseline or after 12 months (*p* > 0.05). Belli et al. (2014) observed a similar trend, noting that the addition of glass fillers did not significantly affect water sorption. We also did not observe any measurable solubility at 24 h. Hence, only the 12-month solubility data have been reported in the current study (Table 1). The solubility data showed that the addition of BNNSs did not significantly affect water solubility. A similar non-significant effect was observed by Belli et al. (2014) using glass-filler-reinforced methacrylic polymer adhesive systems [17]. At such low-filler loadings (≤1 wt%), any effect by the BNNSs may have been too small to be observable.

### 3.3. Flexural Strength

**Figure 4:**
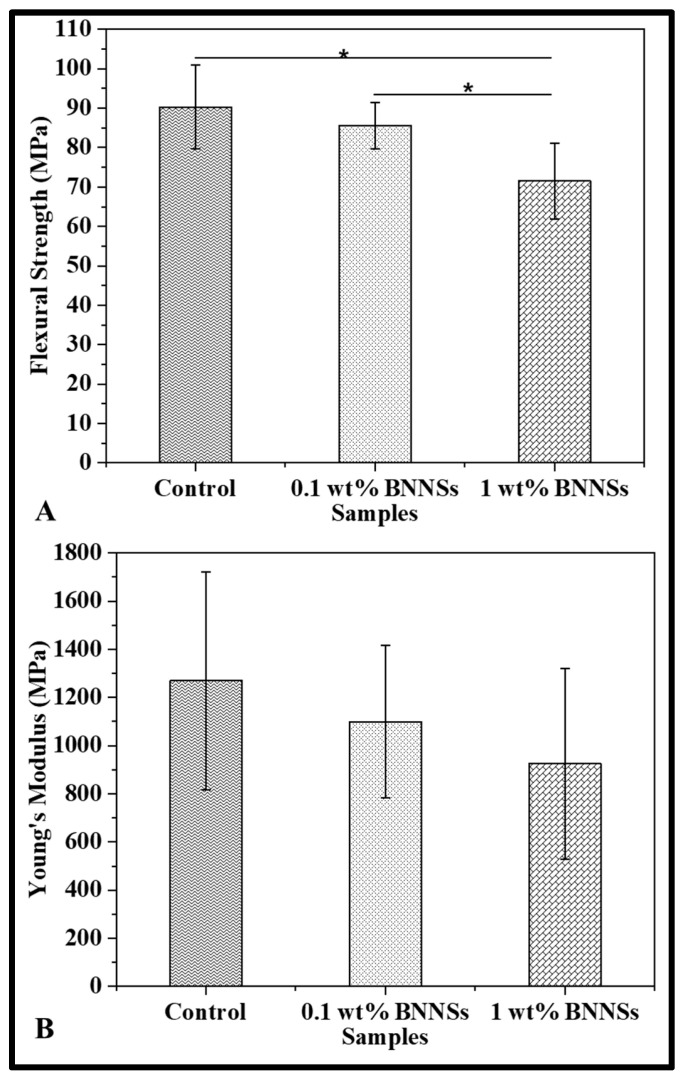
(**A**) Flexural strength and (**B**) Young’s modulus of the adhesive. The data were expressed as mean ± standard deviation. Statistical significance was evaluated for *p* < 0.05 via one-way ANOVA. * Data were statistically significant (*p* < 0.05).

Flexural strength and Young’s modulus are important mechanical properties in evaluating the performance of dental resins and adhesives [5,42,54,55,56]. Mechanical testing results showed that control adhesives had similar flexural strength (90.3 ± 10.6 MPa) to the 0.1 wt% BNNSs (85.6 ± 5.9 MPa) (Figure 4A). Both these groups had significantly higher flexural strengths than the 1 wt% BNNSs group (71.5 ± 9.7 MPa; *p* < 0.05). However, there were no significant differences in moduli among the groups (*p* > 0.05; Figure 4B). Shojai et al. (2010) showed that the addition of hydroxyapatite nanofillers of 5% resulted in a significant reduction in both flexural strength and Young’s modulus (*p* < 0.05). The author reported that the agglomeration of nanofillers at higher loading percentage (≥5%) and the resultant incomplete curing could be the reasons for the significant drop in the strength [5]. Similarly, with the loading of BNNSs at more than 0.1 wt%, there could have been agglomeration of BNNSs resulting in reduced strength, even though the DoC was higher than that of the controls. The lack of a significant decrease in Young’s modulus could be due to the high aspect ratio of the nanosheets increasing modulus in the direction of the flexural stress. 

### 3.4. Morphological Analysis

**Figure 5:**
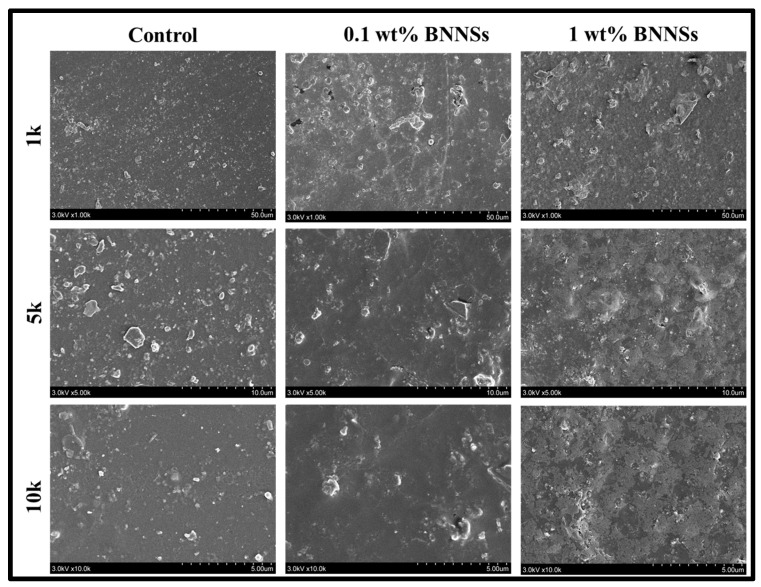
SEM micrographs of the outer surfaces of the samples.

**Figure 6:**
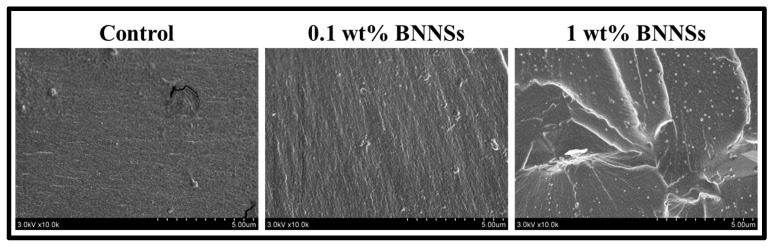
SEM micrographs of the fractured surfaces of the samples.

The effect of the addition of BNNSs on the morphology of the adhesive system was evaluated with SEM. The SEM micrographs of the outer surface revealed that the roughness of the adhesive surface increased with the addition of filler and the increasing loading percentage. However, it did not show significant variation between the control and 0.1 wt% BNNSs samples; nonetheless, the 1 wt% BNNSs sample clearly showed higher roughness (Figure 5). The SEM micrographs of the fractured surface clearly showed that the surface of the control group was smooth, where sample 0.1 wt% BNNSs had a slightly rough surface (Figure 6). The addition of 1 wt% BNNSs has altered the surfaces of adhesives, which could be observed in their roughness. Additionally, as expected, the 0.1 wt% BNNSs addition showed a uniform distribution of BNNSs, as evident from the even surface morphology. On the contrary, the 1 wt% BNNSs addition caused non-uniform distribution and agglomeration of BNNSs, which resulted in an uneven surface with higher roughness. Sadat-Shojai et al. (2009) observed a significant increase in roughness with the addition of hydroxyapatite nanorods into a dental polymeric adhesive system [5]. The fractured surface was observed to be smooth for the control and 0.1 wt% BNNSs group, whereas it was rougher with the increase in filler content, as observed in the 1 wt% BNNSs sample. This could be caused by the cracks which developed at the agglomerated particle surface. A similar observation was reported by Belli et al. (2014) while reinforcing adhesives with different loading percentages of glass fillers [17]. 

### 3.5. Microtensile Bond Strength and Failure Pattern Analysis

**Figure 7:**
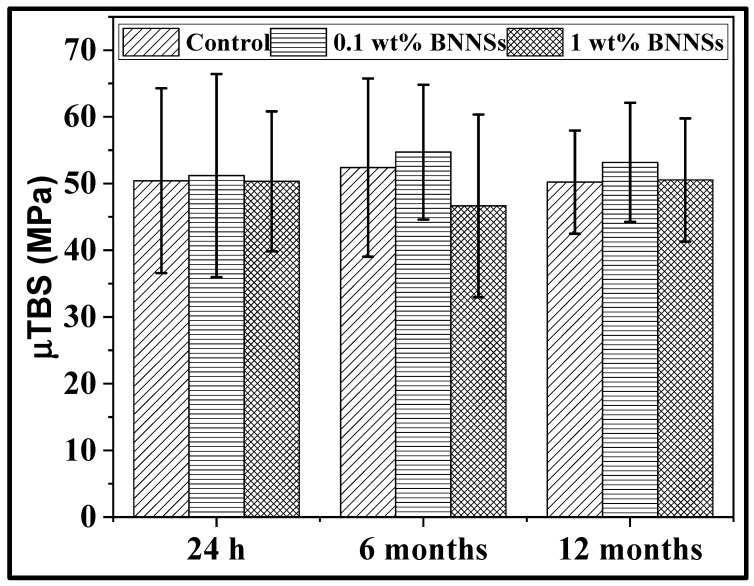
Microtensile bond strength of the adhesives. The data were expressed as mean ± standard deviation. Statistical significance was evaluated for *p* < 0.05 by Two-way ANOVA.

The ultimate performance of an adhesive system is defined by its ability to form a strong and durable bond between the tooth surface and the resins [43]. The current study was conducted at three different time points to assess the durability of the bond: 24 h; 6 months; and 12 months (Figure 7). The baseline study at 24 h showed that bond strengths of control (0 wt% BNNSs) = 50.43 ± 13.86 MPa; 0.1 wt% BNNSs = 51.21 ± 15.23 MPa; and 1 wt% BNNSs = 50.32 ± 10.49 MPa. The bond strengths at 6-month time were 52.39 ± 13.36 MPa, 54.72 ± 10.1 MPa, and 46.66 ± 13.69 MPa, respectively. The bond strengths at 12 months of incubation were 50.22 ± 7.73 MPa, 53.18 ± 8.93 MPa, and 50.54 ± 9.24 MPa, respectively. 

It was reported by I.R. Reynolds that the minimum bond strength of orthodontic adhesives is 6–8 MPa [57,58]. Brantley et al. (2001) reported the existence of an orthodontic adhesive system with shear bond strength varying at a greater range of about 8–30 MPa [59]. The current sample values are comparable or higher than those of the above-mentioned values. There were no differences in bond strength among the groups and among the time points (*p* > 0.05). It has been reported in several studies that the addition of fillers into polymers increase the mechanical properties of adhesives, which, in turn, enhance the bond strength. However, over a certain loading percentage, there could be a reduction in the microtensile bond strength. Menezes et al. (2016) showed that the addition of montmorillonite clay into a polymer adhesive system improved the thermal and mechanical properties up to a loading percentage of 0.2 wt%. However, at greater than 0.2 wt%, the filler did not disperse well, and bond strength did not increase [60]. It is possible that the BNNS loading was too low to provide a significant effect. 

The failure mode analysis showed that the 0.1 wt% BNNSs group had the lowest adhesive failure percentage at all time points (24 h, 6 months, and 12 months), and the 1 wt% BNNSs group had a higher adhesive failure percentage (Table 2). The percentage of adhesive failure decreased in the 6-month study in comparison to baseline (24 h) data. The same had increased by 12 months of incubation. Others have reported that adhesives with fillers had more adhesive failures than cohesive or mixed [51,61,62]. However, Torres-Rosas et al. (2020) reported that the addition of copper nanoparticles improved the mechanical properties of the adhesive and decreased the percentage of adhesive failure. The study claimed that the addition of nanofillers improved the bond strength of the adhesive [19]. Similarly, in this study, the addition of BNNSs up to 0.1 wt% had a slight incremental effect on the bond strength, though the data were insignificant (*p* > 0.05).

## 4. Conclusions

The current study showed that incorporating BNNSs at different loading percentages increased the degree of cure of the adhesive. However, the addition of BNNSs did not influence the water absorption and solubility of the adhesive system. A similar phenomenon was also observed with respect to Young’s modulus. Instead, the addition of high BNNS loading (1 wt%) had a detrimental effect on the flexural strength of the adhesive. Microtensile bond strength and failure mode analysis revealed that adding BNNSs up to 1 wt% did not significantly affect the bond strength or the performance of the adhesive system. However, the current study is limited to in vitro testing, which can provide insight into applying BNNSs as a filler in dental adhesives. Further studies are needed with varying BNNS loading percentages from 0.1 wt% to 1 wt% in order to determine the effect of BNNSs effectively. Additional long-term mechanical tests could be performed to evaluate the system more accurately.

## Figures and Tables

**Table 1:** Water sorption and solubility of the adhesive system.

Samples	Water Sorption (µg/mm^3^)		Water Solubility (µg/mm^3^)
	7 Days	12 Months	
**Control**	103.4 ± 7.2	118.78 ± 6.48	5.54 ± 2.01
**0.1 wt% BNNSs**	101.9 ± 7.5	118.67 ± 7.87	2.62 ± 1.88
**1 wt% BNNSs**	103.3 ± 2.4	119.72 ± 9.63	3.48 ± 2.53

**Table 2:** Failure mode analysis of the specimens with different adhesive systems at different time points (24 h, 6 months, and 12 months).

Time	Samples	Failure Mode Analysis (%)			
		Adhesive	Dentin	Composite	Mixed
24 h	Control	58.82	35.29	5.88	0
	0.1 wt% BNNSs	35.29	58.82	0	5.88
	1 wt% BNNSs	64.71	23.53	0	11.76
6 months	Control	41.18	41.18	11.76	5.88
	0.1 wt% BNNSs	11.76	64.71	23.53	0
	1 wt% BNNSs	47.06	41.18	11.76	0
12 months	Control	47.06	41.18	0	11.76
	0.1 wt% BNNSs	29.41	41.18	17.65	11.76
	1 wt% BNNSs	58.82	23.53	0	17.65

## Data Availability

New data were created and analyzed during this study. These datasets are not publicly available at this time but can be obtained from the corresponding author, Erica C. Teixeira, upon reasonable request.

## References

[1] Degrazia F.W., Leitune V.C.B., Visioli F., Samuel S.M.W., Collares F.M. (2018). Long-term stability of dental adhesive incorporated by boron nitride nanotubes. Dent. Mater..

[2] Sauro S., Pashley D.H. (2016). Strategies to stabilise dentine-bonded interfaces through remineralising operative approaches–State of The Art. Int. J. Adhes. Adhes..

[3] Vaidyanathan T., Vaidyanathan J. (2009). Recent advances in the theory and mechanism of adhesive resin bonding to dentin: A critical review. J. Biomed. Mater. Res. Part B Appl. Biomater. Off. J. Soc. Biomater. Jpn. Soc. Biomater. Aust. Soc. Biomater. Korean Soc. Biomater..

[4] Pashley D.H., Swift E.J. (2008). Dentin bonding. J. Esthet. Restor. Dent. Off. Publ. Am. Acad. Esthet. Dent..

[5] Sadat-Shojai M., Atai M., Nodehi A., Khanlar L.N. (2010). Hydroxyapatite nanorods as novel fillers for improving the properties of dental adhesives: Synthesis and application. Dent. Mater..

[6] Ferracane J.L. (2006). Hygroscopic and hydrolytic effects in dental polymer networks. Dent. Mater..

[7] Nishitani Y., Yoshiyama M., Donnelly A., Agee K., Sword J., Tay F., Pashley D.H. (2006). Effects of resin hydrophilicity on dentin bond strength. J. Dent. Res..

[8] de Andrade e Silva S.M., Carrilho M., Garcia F., Manso A., Alves M., de Carvalho R. (2009). Effect of an additional hydrophilic versus hydrophobic coat on the quality of dentinal sealing provided by two-step etch-and-rinse adhesives. J. Appl. Oral Sci. Rev. FOB.

[9] Ilie N., Serfözö N.E., Prodan D., Diegelmann J., Moldovan M. (2022). Synthesis and performance of experimental resin-based dental adhesives reinforced with functionalized graphene and hydroxyapatite fillers. Mater. Des..

[10] Sanghvi M.R., Tambare O.H., More A.P. (2022). Performance of various fillers in adhesives applications: A review. Polym. Bull..

[11] Leitune V.C.B., Collares F.M., Takimi A., de Lima G.B., Petzhold C.L., Bergmann C.P., Samuel S.M.W. (2013). Niobium pentoxide as a novel filler for dental adhesive resin. J. Dent..

[12] Khosravi K., Mirmohamadi H., Kashani K. (2012). Evaluation of effect of adding silica fillers to adhesive on microleakage of composite restorations in different times. J. Iran. Dent. Assoc..

[13] Giannini M., Mettenburg D., Arrais C., Rueggeberg F.A. (2011). The effect of filler addition on biaxial flexure strength and modulus of commercial dentin bonding systems. Quintessence Int..

[14] Rodríguez H.A., Kriven W.M., Casanova H. (2019). Development of mechanical properties in dental resin composite: Effect of filler size and filler aggregation state. Mater. Sci. Eng. C.

[15] Shinkai K., Taira Y., Suzuki S., Kawashima S., Suzuki M. (2018). Effect of filler size and filler loading on wear of experimental flowable resin composites. J. Appl. Oral Sci..

[16] Raszewski Z., Brząkalski D., Derpeński Ł., Jałbrzykowski M., Przekop R.E. (2022). Aspects and principles of material connections in Restorative dentistry—A comprehensive review. Materials.

[17] Belli R., Kreppel S., Petschelt A., Hornberger H., Boccaccini A.R., Lohbauer U. (2014). Strengthening of dental adhesives via particle reinforcement. J. Mech. Behav. Biomed. Mater..

[18] Carvalho E., De Paula D., Neto D.A., Costa L., Dias D., Feitosa V., Fechine P. (2020). Radiopacity and mechanical properties of dental adhesives with strontium hydroxyapatite nanofillers. J. Mech. Behav. Biomed. Mater..

[19] Torres-Rosas R., Torres-Gómez N., García-Contreras R., Scougall-Vilchis R.J., Domínguez-Díaz L.R., Argueta-Figueroa L. (2020). Copper nanoparticles as nanofillers in an adhesive resin system: An in vitro study. Dent. Med. Probl..

[20] Lezaja M., Jokic B.M., Veljovic D.N., Miletic V. (2016). Shear bond strength to dentine of dental adhesives containing hydroxyapatite nano-fillers. J. Adhes. Sci. Technol..

[21] da Cruz L.B.T., Oliveira M.T., Saraceni C.H.C., Lima A.F. (2019). The influence of nanofillers on the properties of ethanol-solvated and non-solvated dental adhesives. Restor. Dent. Endod..

[22] Lohbauer U., Wagner A., Belli R., Stoetzel C., Hilpert A., Kurland H.-D., Grabow J., Müller F.A. (2010). Zirconia nanoparticles prepared by laser vaporization as fillers for dental adhesives. Acta Biomater..

[23] Frank I., Tanenbaum D.M., van der Zande A.M., McEuen P.L. (2007). Mechanical properties of suspended graphene sheets. J. Vac. Sci. Technol. B Microelectron. Nanometer Struct. Process. Meas. Phenom..

[24] Lucibella M. (2009). This Month is Physics History-October 22, 2004: Discovery of Graphene. Am. Phys. Soc.-APS News.

[25] Bregnocchi A., Zanni E., Uccelletti D., Marra F., Cavallini D., De Angelis F., De Bellis G., Bossù M., Ierardo G., Polimeni A. (2017). Graphene-based dental adhesive with anti-biofilm activity. J. Nanobiotechnol..

[26] Mei L., Wei H., Wenjing C., Xiaokun H. (2017). Graphene oxide-silica composite fillers into the experimental dental adhesives for potential therapy. Med. Res..

[27] Mourad M., Wijnhoven J., Van’t Zand D., van der Beek D., Lekkerkerker H.N. (2006). Gelation versus liquid crystal phase transitions in suspensions of plate-like particles. Philos. Trans. R. Soc. A Math. Phys. Eng. Sci..

[28] Mourad M.C., Petukhov A.V., Vroege G.J., Lekkerkerker H.N. (2010). Lyotropic hexagonal columnar liquid crystals of large colloidal gibbsite platelets. Langmuir.

[29] Van der Kooij F.M., Kassapidou K., Lekkerkerker H.N. (2000). Liquid crystal phase transitions in suspensions of polydisperse plate-like particles. Nature.

[30] Qi-lin X., Zhen-huan L., Xiao-geng T. (2015). The defect-induced fracture behaviors of hexagonal boron-nitride monolayer nanosheets under uniaxial tension. J. Phys. D Appl. Phys..

[31] Singh S.K., Neek-Amal M., Costamagna S., Peeters F. (2013). Thermomechanical properties of a single hexagonal boron nitride sheet. Phys. Rev. B.

[32] Lee B., Lee D., Lee J.H., Ryu H.J., Hong S.H. (2016). Enhancement of toughness and wear resistance in boron nitride nanoplatelet (BNNP) reinforced Si3N4 nanocomposites. Sci. Rep..

[33] Falin A., Cai Q., Santos E.J., Scullion D., Qian D., Zhang R., Yang Z., Huang S., Watanabe K., Taniguchi T. (2017). Mechanical properties of atomically thin boron nitride and the role of interlayer interactions. Nat. Commun..

[34] Chen J., Chen B., Li J., Tong X., Zhao H., Wang L. (2017). Enhancement of mechanical and wear resistance performance in hexagonal boron nitride-reinforced epoxy nanocomposites. Polym. Int..

[35] Sukhorukova I.V., Zhitnyak I.Y., Kovalskii A.M., Matveev A.T., Lebedev O.I., Li X., Gloushankova N.A., Golberg D., Shtansky D.V. (2015). Boron nitride nanoparticles with a petal-like surface as anticancer drug-delivery systems. ACS Appl. Mater. Interfaces.

[36] Mateti S., Wong C.S., Liu Z., Yang W., Li Y., Li L.H., Chen Y. (2018). Biocompatibility of boron nitride nanosheets. Nano Res..

[37] Satsangi N., Rawls H.R., Norling B.K. (2005). Synthesis of low-shrinkage polymerizable methacrylate liquid-crystal monomers. J. Biomed. Mater. Res. Part B Appl. Biomater. Off. J. Soc. Biomater. Jpn. Soc. Biomater. Aust. Soc. Biomater. Korean Soc. Biomater..

[38] Zhi C., Bando Y., Tang C., Kuwahara H., Golberg D. (2009). Large-scale fabrication of boron nitride nanosheets and their utilization in polymeric composites with improved thermal and mechanical properties. Adv. Mater..

[39] Degrazia F.W., Leitune V.C.B., Samuel S.M.W., Collares F.M. (2017). Boron nitride nanotubes as novel fillers for improving the properties of dental adhesives. J. Dent..

[40] Bohns F.R., Degrazia F.W., de Souza Balbinot G., Leitune V.C.B., Samuel S.M.W., García-Esparza M.A., Sauro S., Collares F.M. (2019). Boron nitride nanotubes as filler for resin-based dental sealants. Sci. Rep..

[41] Sarikaya R., Song L., Ye Q., Misra A., Tamerler C., Spencer P. (2020). Evolution of network structure and mechanical properties in autonomous-strengthening dental adhesive. Polymers.

[42] Atai M., Nekoomanesh M., Hashemi S., Amani S. (2004). Physical and mechanical properties of an experimental dental composite based on a new monomer. Dent. Mater..

[43] Solhi L., Atai M., Nodehi A., Imani M., Ghaemi A., Khosravi K. (2012). Poly (acrylic acid) grafted montmorillonite as novel fillers for dental adhesives: Synthesis, characterization and properties of the adhesive. Dent. Mater..

[44] (2003). Dental Materials—Testing of Adhesion to Tooth Structure.

[45] Malacarne J., Carvalho R.M., de Goes M.F., Svizero N., Pashley D.H., Tay F.R., Yiu C.K., de Oliveira Carrilho M.R. (2006). Water sorption/solubility of dental adhesive resins. Dent. Mater..

[46] Malacarne-Zanon J., Pashley D.H., Agee K.A., Foulger S., Alves M.C., Breschi L., Cadenaro M., Garcia F.P., Carrilho M.R. (2009). Effects of ethanol addition on the water sorption/solubility and percent conversion of comonomers in model dental adhesives. Dent. Mater..

[47] Marghalani H.Y. (2012). Sorption and solubility characteristics of self-adhesive resin cements. Dent. Mater..

[48] Bin-Shuwaish M.S., Maawadh A.M., Al-Hamdan R.S., Alresayes S., Ali T., Almutairi B., Vohra F., Abduljabbar T. (2020). Influence of graphene oxide filler content on the dentin bond integrity, degree of conversion and bond strength of experimental adhesive. A SEM, micro-raman, FTIR and microtensile study. Mater. Res. Express.

[49] Carneiro K.K., Meier M.M., Santos C.C.d., Maciel A.P., Carvalho C.N., Bauer J. (2016). Adhesives doped with bioactive niobophosphate micro-filler: Degree of conversion and microtensile bond strength. Braz. Dent. J..

[50] Aguiar T.R., De Oliveira M., Arrais C.A., Ambrosano G.M., Rueggeberg F., Giannini M. (2015). The effect of photopolymerization on the degree of conversion, polymerization kinetic, biaxial flexure strength, and modulus of self-adhesive resin cements. J. Prosthet. Dent..

[51] AlRefeai M.H., AlHamdan E.M., Al-Saleh S., Farooq I., Abrar E., Vohra F., Abduljabbar T. (2021). Assessment of bond integrity, durability, and degree of conversion of a calcium fluoride reinforced dentin adhesive. Polymers.

[52] Ito S., Hashimoto M., Wadgaonkar B., Svizero N., Carvalho R.M., Yiu C., Rueggeberg F.A., Foulger S., Saito T., Nishitani Y. (2005). Effects of resin hydrophilicity on water sorption and changes in modulus of elasticity. Biomaterials.

[53] Durner J., Spahl W., Zaspel J., Schweikl H., Hickel R., Reichl F.-X. (2010). Eluted substances from unpolymerized and polymerized dental restorative materials and their Nernst partition coefficient. Dent. Mater..

[54] Zandinejad A., Atai M., Pahlevan A. (2006). The effect of ceramic and porous fillers on the mechanical properties of experimental dental composites. Dent. Mater..

[55] Tanimoto Y., Kitagawa T., Aida M., Nishiyama N. (2006). Experimental and computational approach for evaluating the mechanical characteristics of dental composite resins with various filler sizes. Acta Biomater..

[56] Liu Y., Tan Y., Lei T., Xiang Q., Han Y., Huang B. (2009). Effect of porous glass–ceramic fillers on mechanical properties of light-cured dental resin composites. Dent. Mater..

[57] Reynolds I. (1975). A review of direct orthodontic bonding. Br. J. Orthod..

[58] Reynolds I. (1975). Composite filling materials as adhesives in orthodontics. Br. Dent. J..

[59] Brantley W.A., Eliades T. (2001). Orthodontic materials: Scientific and clinical aspects. Am. J. Orthod. Dentofac. Orthop..

[60] Menezes L.R.d., Silva E.O.d. (2016). The use of montmorillonite clays as reinforcing fillers for dental adhesives. Mater. Res..

[61] Al-Hamdan R.S., Almutairi B., Kattan H.F., Alsuwailem N.A., Farooq I., Vohra F., Abduljabbar T. (2020). Influence of hydroxyapatite nanospheres in dentin adhesive on the dentin bond integrity and degree of conversion: A scanning electron microscopy (SEM), raman, fourier transform-infrared (FTIR), and microtensile study. Polymers.

[62] AlFawaz Y.F., Almutairi B., Kattan H.F., Zafar M.S., Farooq I., Naseem M., Vohra F., Abduljabbar T. (2020). Dentin bond integrity of hydroxyapatite containing resin adhesive enhanced with graphene oxide nano-particles—An SEM, EDX, micro-Raman, and microtensile bond strength study. Polymers.

